# Bioprocess Development and Characterization of a ^13^C-Labeled Hybrid Bispecific Antibody Produced in *Escherichia coli*

**DOI:** 10.3390/antib12010016

**Published:** 2023-02-14

**Authors:** Aaron T. Wecksler, Victor Lundin, Ambrose J. Williams, Karthik Veeravalli, Dorothea E. Reilly, Sung-Hye Grieco

**Affiliations:** 1 Protein Analytical Chemistry, Genentech Inc., South San Francisco, CA 94080, USA; 2 Purification Development, Genentech Inc., South San Francisco, CA 94080, USA; 3 Cell Culture and Bioprocess Operations, Genentech Inc., South San Francisco, CA 94080, USA

**Keywords:** biotherapeutic proteins, protein isotopic labeling, ambr250 bioreactors, ^13^C-celtone, ^13^C-labeled glucose, knob-into-hole

## Abstract

Monoclonal antibodies (mAbs) are highly efficacious therapeutics; however, due to their large, dynamic nature, structural perturbations and regional modifications are often difficult to study. Moreover, the homodimeric, symmetrical nature of mAbs makes it difficult to elucidate which heavy chain (HC)-light chain (LC) pairs are responsible for any structural changes, stability concerns, and/or site-specific modifications. Isotopic labeling is an attractive means for selectively incorporating atoms with known mass differences to enable identification/monitoring using techniques such as mass spectrometry (MS) and nuclear magnetic resonance (NMR). However, the isotopic incorporation of atoms into proteins is typically incomplete. Here we present a strategy for incorporating ^13^C-labeling of half antibodies using an *Escherichia coli* fermentation system. Unlike previous attempts to generate isotopically labeled mAbs, we provide an industry-relevant, high cell density process that yielded >99% ^13^C-incorporation using ^13^C-glucose and ^13^C-celtone. The isotopic incorporation was performed on a half antibody designed with knob-into-hole technology to enable assembly with its native (naturally abundant) counterpart to generate a hybrid bispecific (BsAb) molecule. This work is intended to provide a framework for producing full-length antibodies, of which half are isotopically labeled, in order to study the individual HC-LC pairs.

## 1. Introduction

Monoclonal antibodies (mAbs) represent the largest contingent of approved biotherapeutics on the market [1,2]. MAbs are ideal as a therapeutic due to their high specificity, long serum half-life, and ability to evade the human immune system [3]. The structure of a conventional mAb consists of a homodimeric, heavy chain (HC)-light chain (LC) pair that is held together by interchain disulfide bonds, resulting in the canonical Y-shaped structure [4]. However, structural analysis of mAbs can be difficult due to the size of the protein and the highly dynamic nature of the variable antigen binding region (Fab) and the conserved fragment crystallizable region (Fc) [5]. 

The different approaches used for mAb structural elucidation include spectroscopic methods [6], calorimetry [7], nuclear magnetic resonance [8], crystallography [9], electron microscopy [10], and mass spectrometry (MS)-based techniques [11]. Regardless of the technology used for structural characterizations, all of the methods face the inherent challenge of the homodimeric symmetry of antibodies. It is nearly impossible to determine which side of the antibody correlates with the structural analysis, and this becomes increasingly challenging with MS-based technologies in which there are identical sequences in the HC-LC pairs. 

A potential strategy for deciphering which HC-LC pair is involved in structural observations is to leverage isotopic incorporation. Isotopically labeled proteins have been used for decades for a myriad of applications such as in vivo tracing [12], radiolabeled therapies [13], NMR [14], and liquid chromatography (LC)-MS absolute quantification [15]. There has also been work on isotopically labeled antibodies; however, the previous strategies have been mostly focused on the random incorporation of atom isotopes [16] or selective incorporation of isotopically labeled amino acids [17]. Depending on the application, these strategies have utility for certain types of analytical characterization, but the challenge with the homodimeric structure of the antibody remains. 

In this report, we set out to develop a strategy for generating a mAb construct in which the individual HC-LC pairs can be deciphered using MS-based technologies. This strategy combines biotechnology industry-relevant fermentation processes and knob-into-hole technology [18] to generate an *E. coli*-derived hybrid bispecific antibody (hBsAb) with heterodimeric ^13^C-isotopic incorporation. This work is the first-of-its-kind to demonstrate the feasibility of generating an hBsAb with complete ^13^C-incorporation in only one of the HC-LC pairs. Given that the incorporation of the starting ^13^C-glucose and ^13^C-celtone is claimed to be 99% and 98%+, respectively, the incorporation from the strategy presented herein, achieved a 100% of the expected incorporation. This type of hBsAb construct may enable structural characterization of the individual HC-LC pairs for MS-based applications such as epitope mapping, Fc-receptor interactions, degradation susceptibilities, and the potential for absolute quantitation of post-translational and sequence variant modifications. 

## 2. Methods

### 2.1. Bacterial Strain and Plasmids

An in-house genetically modified *Escherichia coli* (*E. coli*) strain constructed from W3110 [19] was used for antibody expression. Expression cassettes coding for light and heavy chains of half antibodies, hAb1 and hAb2, were cloned into pBR322 at the EcoRI site [20]. hAb1 and hAb2 consist of one light and one heavy chain each and are two versions of the same half antibody with knob and hole mutations in the CH3 domain, respectively (hAb1 has the knob mutation while hAb2 has the hole mutation) [18]. The *phoA* promoter was used to drive expression of antibody-coding genes, and the STII signal sequence with a silent codon variant preceded the coding sequence of both light and heavy chains [21]. The chaperone-coding genes, *dsbA*, *dsbC*, and *fkpA*, were first assembled into a single fragment using overlap PCR, with the *tac* promoter driving expression of *dsbA* and *dsbC* and the *phoA* promoter driving expression of *fkpA*. The resulting fragment was then cloned into the antibody expression plasmid in the XhoI and AvrII sites. 

### 2.2. Fermentations

Primary inoculum cultures were inoculated from a frozen vial lot of *E. coli* containing the appropriate plasmid. Cultures were grown in Falcon 50 mL conical centrifuge tubes (ThermoFisher Scientific, Waltam, MA, USA) with a working volume of 10 mL for 16 hr at 300 RPM and 30 °C. The entire primary inoculum culture was used to inoculate the production culture fermentations, which were performed using ambr250 bioreactors (Sartorius Stedim, Concord, CA, USA). Production culture fermentations were performed at pH 6.7, 34 °C, 1170 RPM, and an airflow of 2 vvm. At a cell density of approximately 150 OD_550_, the temperature was ramped down to 25 °C over a duration of 1 h. Immediately following the start of the temperature ramp (at a cell density of approximately 150 OD_550_), agitation was ramped down to achieve an oxygen uptake rate target of 2.3 mmol/L-min over a duration of 2 h. Production fermentations were performed for 72 h. 

Separate fermentations were used for the production of the two half antibodies, hAb1 and hAb2. Media and feeds used for primary inoculum cultures and production fermentations were as previously described [21]. For experiments using Celtone^®^ base (Cambridge isotope laboratories, Inc., Andover, MA, USA, referred to as Celtone from here on), modifications were as follows: tryptone (10 g/L) and yeast extract (5 g/L) in the primary inoculum culture medium were replaced with Celtone (Cambridge isotope laboratories, Inc., Andover, MA, USA) at 15 g/L. NZ Soy BL4 (29 g/L) and yeast extract (14.3 g/L) in the production culture medium were replaced with Celtone (45 g/L). No methionine was fed during the fermentations. For ^13^C-labeling experiments, ^13^C-labeled glucose (Cambridge isotope laboratories, Inc., Andover, MA, USA) and ^13^C-labeled Celtone (Cambridge isotope laboratories, Inc., Andover, MA, USA) were used. 

### 2.3. Analytical Measurements for Monitoring Fermentation

Product titer was measured as described previously [22]. OD550 was measured using a GENESYS 20 visible spectrophotometer (ThermoFisher Scientific, Waltam, MA, USA) by measuring absorbance at 550 nm. Phosphate levels were measured using the COBAS Integra 400 (Roche Diagnostics, Penzberg, Germany) according to published methods [23]. 

### 2.4. Downstream Processing

At the conclusion of fermentation, half-antibodies were recovered from harvested whole-cell broth, purified, and assembled into bispecific antibodies using a previously described method [24]. Briefly: *E. coli* cells were homogenized using a microfluidizer, and the homogenate was flocculated with the addition of the poly-cation polyethyleneimine and clarified by centrifugation. Half-antibodies were captured from the clarified centrate using Protein A affinity chromatography. Knob and Hole half-antibodies were combined in a 1:1 molar ratio and assembled into bispecific antibodies by the addition of L-reduced Glutathione, to a final concentration of 5 mM, at pH 8.5 and 37 °C for 22.5 h. Following assembly, the reaction mixture was adjusted to pH 5 and polished by cation exchange chromatography using a POROS XS column with a gradient elution of increasing sodium acetate concentration. A mock pool was created from fractions containing the main peak, and this was used for subsequent analysis.

### 2.5. Mass Spectrometry Analysis and Determination of % ^13^C-Incorporation

Intact and reduced mass spectrometry (MS) was performed to measure the intact and reduced forms of the natural abundant and ^13^C-labeled half antibodies as previously described [25], and data analysis was performed using the MassHunter Software Suite v. B.06.00 (Agilent, Santa Clara, CA, USA). The % ^13^C-incorporation was determined by calculating the difference in the predicted average mass vs. the predicted average mass shift based on the number for carbons (assuming an average mass shift of 1 Da/carbon atom).

% ^13^C-Incorporation = 100 − [100 × ((Mass_(Predicted)_ − Mass_(Observed)_)/(Number of Carbons × 0.99))]

Peptide mapping was performed and analyzed using the Byos^®^ Software Suite (PMI) v.4.2 (Protein Metric Inc., Cupertino, CA, USA) as previously published [26].

## 3. Results

### 3.1. Fermentation

*E. coli* fermentation media used in-house consists of a yeast extract, tryptone, and NZ Soy BL4 to support growth to high cell densities and the production of recombinant proteins. Yeast extract and tryptone were replaced with Celtone in the primary inoculum culture media to mimic the chemically defined medium used in biotherapeutic production, and its impact on growth was evaluated. Similarly, yeast extract and NZ Soy BL4 were replaced with Celtone in the production culture media, and its impact on growth and titer was evaluated.

**Figure 1:**
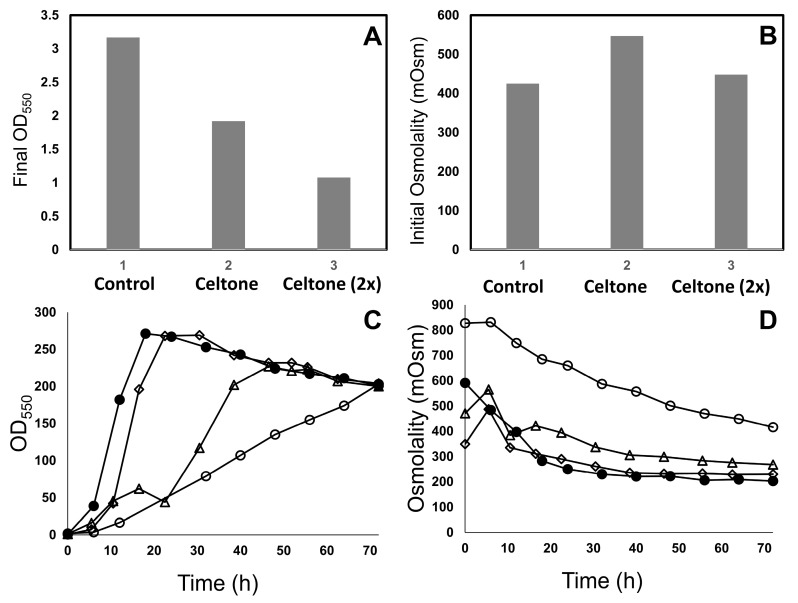
End-of-run growth (**A**) and initial osmolality (**B**) of *E. coli* cultures expressing hAb1 grown in primary inoculum culture media containing tryptone and yeast extract (control), unlabeled Celtone (Celtone), and unlabeled Celtone 2-fold dilution (Celtone 2-fold). Results shown are from *n* = 1 experiment. Growth (**C**) and osmolality (**D**) of *E. coli* cultures expressing hAb1 grown in production fermentation media containing NZ Soy BL4 and yeast extract (filled circles), unlabeled Celtone (open circles), unlabeled Celtone 2-fold dilution (open triangles), and unlabeled Celtone 4-fold dilution (open diamonds). Results shown are from *n* = 1 experiment.

Celtone had an impact on the growth of primary inoculum cultures, as the final OD_550_ was approximately 40% lower than cultures grown in control medium containing tryptone and yeast extract (Figure 1A). Primary inoculum culture medium containing Celtone had a 29% higher initial osmolality compared to control medium (Figure 1B). To evaluate whether higher osmolality impacted growth, Celtone was diluted 2-fold (final concentration of 7.5 g/L) and used to prepare the primary inoculum growth media. Although the osmolality of 2-fold-diluted Celtone was comparable to the control medium (Figure 1B), the final OD_550_ was significantly lower compared to both the control and undiluted Celtone medium cases (Figure 1A). These results indicate that higher osmolality in undiluted Celtone is unlikely to be the cause of lower growth in primary inoculum culture. The lower growth is likely due to the absence and/or lower than optimal concentration of one or more media components in Celtone compared to control (tryptone and yeast extract) media. For subsequent production culture fermentations with Celtone, primary cultures grown in undiluted Celtone were used as the inoculum source. 

Fermentations performed with Celtone in the production medium also resulted in poor growth compared to the control medium containing NZ Soy BL4 and yeast extract (Figure 1C). Production culture medium containing Celtone had a 40% higher initial osmolality compared to the control medium (Figure 1B). To evaluate whether higher osmolality impacted growth, fermentations were performed with diluted Celtone (2-fold and 4-fold at 22.5 g/L and 11.25 g/L final concentration, respectively) in the medium. While a 2-fold dilution of Celtone also resulted in slower growth compared to the control fermentation (NZ Soy BL4 and yeast extract), a 4-fold dilution of Celtone resulted in a slightly longer lag phase but a similar exponential phase growth rate compared to the fermentation using the control medium. Cell densities at the end of fermentation were similar for all cases. For subsequent experiments with ^13^C-labeled Celtone, a 4-fold dilution of Celtone (11.25 g/L final concentration) was used in the production medium.

**Figure 2:**
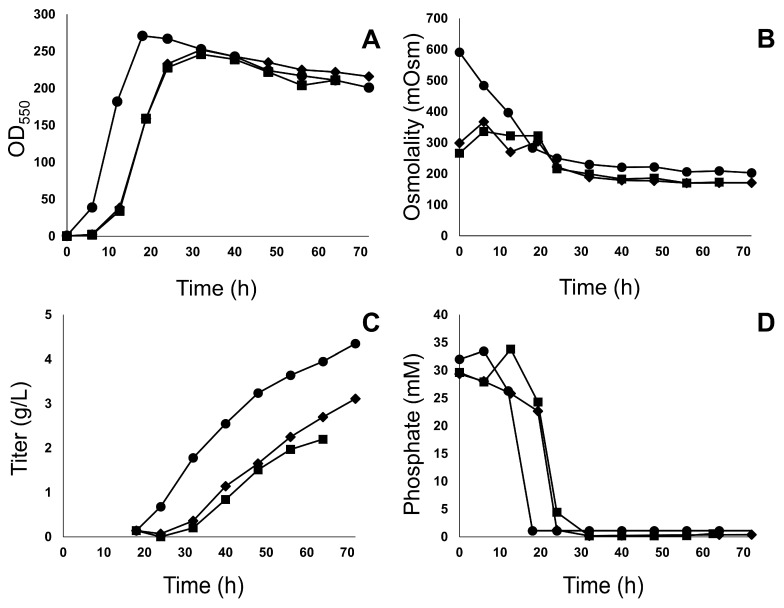
Growth (**A**), osmolality (**B**), titer (**C**), and phosphate depletion (**D**) of *E. coli* cultures expressing hAb1 grown in production fermentation media containing NZ Soy BL4 and yeast extract (filled circles), unlabeled Celtone 4-fold dilution (filled diamonds), and *E. coli* cultures expressing hAb2 grown in media containing ^13^C-labeled Celtone 4-fold dilution (filled squares). Results shown are from *n* = 1 experiment. Note: ^13^C-labeled fermentation was terminated at 64 h due to operational challenges.

The hAb2 production fermentation performed with ^13^C-labeled Celtone (4-fold diluted) in the medium had comparable growth to the hAb1 production fermentation with unlabeled Celtone (4-fold diluted) in the medium (Figure 2A). Both fermentations had a longer lag phase and slightly slower growth compared to the hAb1 production fermentation using the control medium (NZ Soy BL4 and yeast extract) (Figure 2A). As expected, the initial osmolality of the Celtone fermentations was 50% lower than control fermentations (Figure 2B). End-of-fermentation product titers for the unlabeled Celtone and ^13^C-labeled Celtone cases were 30% and 50% lower, respectively, compared to the production fermentation using control medium (Figure 2C). Longer lag and slower growth in the two production fermentations using Celtone resulted in a delayed in phosphate depletion (Figure 2D) and consequently lower product titers compared to control fermentation. 

### 3.2. Mass Spectrometry Analysis and % ^13^C-Incorporation

**Figure 3:**
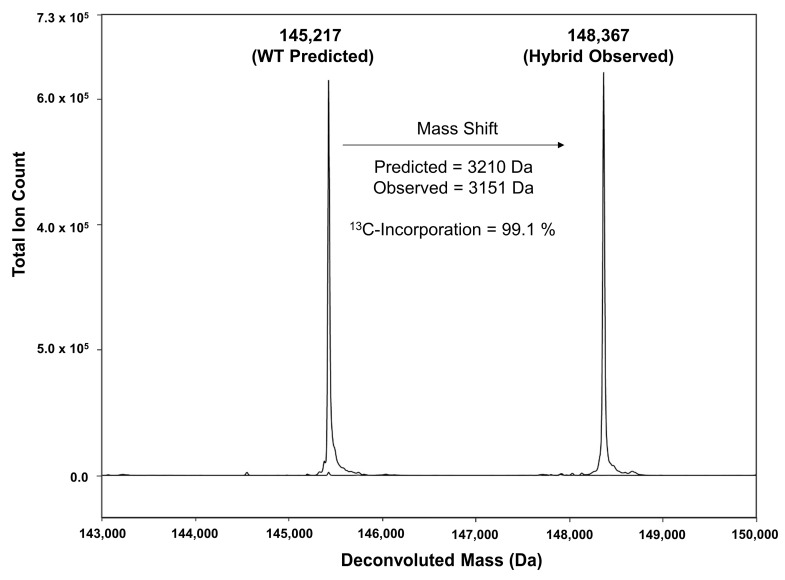
Intact mass analysis of the hybrid molecule (**right side**) compared to the predicted WT molecule (**left side**).

The efficiency of ^13^C labeling was determined by measuring the average masses for the intact and reduced forms of the hole-hAb construct and comparing the observed masses to the theoretical based on the number of carbons within the hole-hAb. As seen in Table 1, the ^13^C-incorporation was observed to be >99% for the hole-hAb in both the intact half-Ab and the reduced HC and LC. The assembled knob (natural abundance)-into-hole (isotopically labeled) to construct the hBsAb resulted in a molecule with the expected mass shift based with >99% ^13^C-incorporation (Table 1, Figure 3). 

## 4. Discussion and Conclusions

Peptide-level characterization of the individual HC-LC pairs of biotherapeutic mAbs using MS-based technologies is nearly impossible due to their inherent homodimeric structure. Novel bispecific constructs such as knob-into-hole [18] and CrossMAbs [27] can provide a framework for characterizing peptides from different HC-LC pairs if used for a monospecific antibody, but this characterization is limited to the peptides containing the residues subjected to mutagenesis. Moreover, bispecific-like constructs are typically designed with the intent to limit the number of mutations in order to avoid undesirable consequences such as protein stability, Fc-receptor function, and immunogenicity concerns. Thus, while bispecific-like constructs may have differences at the peptide level if used for monospecific antibodies, the number of different peptides between the individual half-Abs is typically minimal with respect to the overall sequence identity. In this report, we provide a biotechnologically relevant fermentation framework for generating half-Abs with nearly full ^13^C-incorporation for the specific purpose of characterization of the individual HC-LC pairs using MS-based bottom-up technologies. We acknowledge that the *E. coli* fermentation does have limitations with regards to important post-translational modifications such as glycans, as well as an estimated production cost of approximately 8x that of a standard IgG. Nonetheless, the ability to generate a hybrid bispecific construct with a natural abundance and isotopically labeled half-Abs has the potential to significantly advance our ability to study the individual arms of the antibody’s homodimer structure. 

## Figures and Tables

**Table 1:** Mass spectrometry analysis and determination of % ^13^C-incorporation.

	WT Mass Predicted (Da)	Number of Carbons	Expected Mass Shift (Da)	^13^C-labeling Mass Predicted (Da)	Mass Observed (Da)	^13^C-Incorporation Calculated (%)
hBsAb	145,217	3210	3178	148,395	148,367	99.1
Knob-hAb	72,711	---	---	---	72,712	---
Hole-hAb (Labeled)	72,506	3210	3178	75,684	75,665	99.4
Knob-HC	48,912	---		---	48,912	---
Hole-HC (labeled)	48,707	2169	2147	50,854	50,835	99.1
Knob-LC	23,815	---	---	---	23,815	---
Hole-LC (labeled)	23,815	1041	1031	24,846	24,837	99.1

## Data Availability

Data is contained within the article.
